# Flu and Tdap Maternal Immunization Hesitancy in Times of COVID-19: An Italian Survey on Multiethnic Sample

**DOI:** 10.3390/vaccines9101107

**Published:** 2021-09-29

**Authors:** Anna Franca Cavaliere, Simona Zaami, Marta Pallottini, Federica Perelli, Annalisa Vidiri, Enrico Marinelli, Gianluca Straface, Fabrizio Signore, Giovanni Scambia, Laura Marchi

**Affiliations:** 1 Obstetrics and Gynaecology Unit, Santo Stefano Hospital Prato, AUSL Toscana Centro, Via Suor Niccolina Infermiera, 20/22, 59100 Prato, Italy; annafranca.cavaliere@uslcentro.toscana.it (A.F.C.); marta.pallottini@uslcentro.toscana.it (M.P.); laura.marchi@uslcentro.toscana.it (L.M.); 2 Department of Anatomical, Histological, Forensic and Orthopedic Sciences, “Sapienza” University of Rome, 00185 Rome, Italy; enrico.marinelli@uniroma1.it; 3 Obstetrics and Gynecology Unit, Santa Maria Annunziata Hospital, AUSL Toscana Centro, Via Antella, 58, 50012 Firenze, Italy; federica.perelli@uslcentro.toscana.it; 4 Department of Woman and Child Health, Fondazione Policlinico Universitario A. Gemelli IRCCS, Università Cattolica del Sacro Cuore, Via della Pineta Sacchetti, 217, 00168 Roma, Italy; annalisavidiri@gmail.com (A.V.); giovanni.scambia@policlinicogemelli.it (G.S.); 5 Obstetrics and Gynecology Unit, Abano Terme Polyclinic, Abano, Piazza Cristoforo Colombo, 1, 35031 Abano Terme, Italy; glstraface@gmail.com; 6 Obstetrics and Gynecology Unit, Santo Eugenio Hospital, ASL Roma 2, Piazzale dell’Umanesimo, 10, 00144 Roma, Italy; fabrizio.signore@aslroma2.it

**Keywords:** COVID-19 vaccine, Tdap, influenza vaccine, maternally acquired immunity, pregnancy

## Abstract

Background: Tdap and flu immunization in pregnancy has been proven to be both effective and safe. Despite this, the vaccination rate in pregnant women is low in Italy. The COVID-19 pandemic has focused the attention of public opinion on communicable diseases, underlining the importance of primary prevention measures such as vaccination. We conducted a survey to investigate the behavior of pregnant women during the COVID-19 pandemic regarding maternal immunization to identify the reasons for vaccine hesitancy in order to overcome them. The new challenge is COVID-19 vaccination in pregnancy, and preliminary data show hesitancy towards it. Our analysis may be useful to improve immunization in the pregnant population, including through the COVID-19 vaccine. Methods: A targeted survey was performed in Italy including 520 women who experienced in the first trimester of pregnancy, prior to the novel coronavirus spread, the 2019–2020 influenza vaccination campaign and the Tdap vaccine recommendation in the third trimester during the COVID pandemic. They represent a unique model to investigate if the new coronavirus outbreak might have changed attitudes towards vaccination in pregnancy in the same patients. Data were collected from a self-completed paper questionnaire. Descriptive statistics were calculated and percentages were compared using the chi-2 test or Fisher’s exact test. Results: We obtained data from 195 of the 520 women who gave birth during the inclusion period; 325 cases declined to participate in the survey. A total of 8.7% (17 cases) performed flu vaccination in the first trimester of pregnancy (pre-COVID era), 50.8% (99 cases) accepted Tdap immunization during their third trimester of gestation (COVID-19 pandemic) and 6.7% (13 cases) received both vaccines during pregnancy. For both the flu and Tdap shots, pregnant patients were more likely to accept the vaccines if they were recommended by a healthcare provider, whereas the main reason not to be vaccinated was the lack of such a recommendation. Conclusions: Our survey shows that the COVID-19 experience, which has raised awareness as to the role of vaccines in preventable diseases, may positively change attitudes toward immunization in pregnancy. Vaccination must be recommended to all pregnant women and organized during routine prenatal care as an important element for the prevention of communicable diseases. Vaccination hesitancy can be minimized through consistent recommendation to all pregnant women offered by obstetric staff during routine prenatal care. This approach is likely to be effective in terms of building trust in flu and Tdpa immunization among pregnant women, as well as to avoid unjustified hesitancy towards the more recent COVID-19 vaccines.

## 1. Introduction

Maternal immunization has been proven safe and effective in protecting the mother and/or the offspring from the complications of pertussis and influenza [1]. Passive immunity provided through maternal Tdap (Tetanus, diphtheria, acellular pertussis) vaccination is the main measure that protects infants from pertussis during their first months of life, when they are not able to obtain an optimal vaccine response and are at risk of severe and potentially fatal disease [2]. Influenza vaccination, in addition to eliciting antibody response in the newborn during the first weeks of life, protects the mother and the pregnancy from influenza-related complications [1] (maternal pneumonia, preterm birth, low birthweight). The reviews of several years of surveillance revealed no data that could raise concerns about Tdap and influenza vaccines, either for the fetus or the mother [3,4]. Despite all such evidence, the vaccination rate in pregnant women is globally disappointingly low [5]. In Italy, the National Immunization Plan includes as part of the free routine care of pregnancy both the Tdap vaccination during the third trimester of each pregnancy, in addition to the flu vaccination for all pregnant women during the epidemic season [6,7,8,9]. Despite this, national vaccination coverage among Italian pregnant women is still negligible [10,11,12,13] and even lower than that reported in English-speaking countries. The COVID-19 pandemic has posed a major challenge for healthcare services [14] and has shifted the attention of public opinion on the dangers of communicable diseases, therefore renewing interest and confidence in preventive measures, especially vaccines’ immunization. The offer of COVID-19 vaccines during pregnancy (and/or lactation) is currently supported by the Center for Disease Control [15], the American College of Obstetricians and Gynecologists [16], the Society for Maternal–Fetal Medicine [17] and the Royal College Of Obstetricians and Gynaecologists [18]. This guidance was endorsed by the Società Italiana di Ginecologia ed Ostetricia [19,20]. However, COVID-19 vaccine uptake in pregnant women may be a critical challenge, due to both maternal hesitancy and evolving information offered by healthcare staff [21]. The aim of this survey was to investigate vaccine uptake or hesitancy and their determinants in a group of pregnant women that experienced their first half of pregnancy (pre-COVID pandemic) during the 2019–2020 influenza seasonal epidemic and their third trimester (recommended trimester to perform Tdap immunization) during the novel coronavirus spread. They represent a unique model to investigate if the SARS-CoV-2 outbreak might have changed attitudes towards vaccination in the same pregnant patients. We consider these data useful to maximize SARS-CoV-2 vaccination uptake in the pregnant population.

## 2. Materials and Methods

### 2.1. Study Design and Sampling Method

We conducted a survey in 520 women who had given birth from the beginning of June to the end of August 2020.

Data were collected from a self-administered paper questionnaire offered by clinical staff to all eligible women during their postpartum hospitalization. Questionnaire completion took approximately 15–20 min.

For all participants, written consent was obtained. Both the questionnaire and the informed consent form are available as Supplemental File S1.

### 2.2. Survey Design

The outcome of interest was Tdap vaccination uptake during the COVID-19 pandemic, compared to flu vaccination uptake during the 2019–2020 seasonal influenza epidemic (pre-COVID pandemic) in the same patients. Data reported by pregnant women as part of a self-completed questionnaire (see Supplemental File S1: Survey) included:

- Maternal sociodemographic characteristics;
- Maternal medical history;
- Place and healthcare worker providing prenatal care;
- Perceived reliability of healthcare providers, plans about following national immunization schedule for the newborn;
- Vaccination uptake, main reasons to be vaccinated or not, profession of the healthcare worker recommending the vaccination;
- Maternal knowledge about pertussis and influenza and their vaccines;
- Influence of the COVID-19 pandemic on vaccination.

Each section of the questionnaire included a set of items, and the respondents were asked to choose a predefined answer listed after a question or statement.

Because Chinese and Urdu women represent a significant percentage of pregnant patients who give birth at our hospital, the questionnaire was translated in Chinese and Urdu to include these ethnic minorities.

An information sheet explaining the study was supplied (see Supplemental File S2: Survey information sheet).

### 2.3. Statistical Analysis

For descriptive statistics, frequencies, percentages and averages with standard deviations (SD) were calculated for each question.

Percentages were compared using the chi-2 test or Fisher’s exact test, depending on the number of individuals. The difference was considered significant if *p* < 0.05. The analyses were performed with SPSS software version 24.

## 3. Results

During the inclusion period, 520 deliveries were recorded. A total of 195 women (37.5%) completed and returned the questionnaire and were therefore included in the analysis.

The sociodemographic characteristics of the women included are shown in Table 1. The majority (70.2%) of the women were Italian; however, almost one third of the participants were of a different ethnicity, mainly Chinese (11.8%). More than half (54.3%) of the women were multiparous; 80.5% of them had a medium–high level of education, and 66.7% were in employment.

Eighty-four (43%) women had a pre-existing comorbidity or experienced a pregnancy complication. Almost half (42%) underwent at least one prenatal visit every month. A total of 152 (80%) women received prenatal care at a public hospital outpatient clinic/counselling center, mainly (169 cases, 87%) by a gynecologist–obstetrician. A total of 186 (95.4%) women declared they planned to have their newborns vaccinated following the national schedules for immunization in childhood, only three patients stated they would not adhere to this schedule and only six stated that they needed to further consider this choice (total nine patients—4.6%). When asked about the reliability of their healthcare providers, the majority of women answered they trusted the information received from their gynecologist/midwife (chosen 173 times).

With regard to flu immunization, only 17 patients (8.7%) reported they had been vaccinated—during the pre-pandemic period.

**Figure 1:**
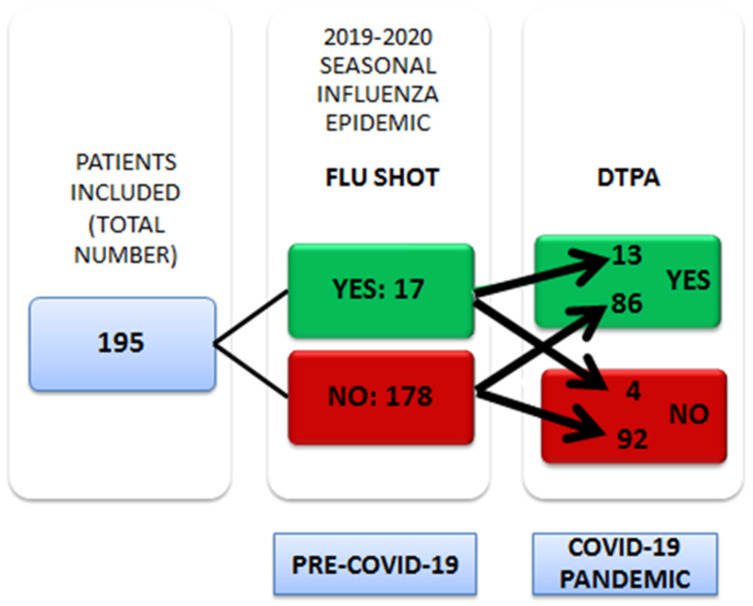
Trend of the uptake of immunization in pregnancy before and during the COVID-19 pandemic in our sample.

As far as pertussis immunization is concerned, more than half of the same women questioned (99 cases, 50.8%) reported they had been vaccinated—during the COVID-19 pandemic. Only 13 patients received both vaccines during pregnancy. The trend of the uptake of immunization in pregnancy before and during the COVID-19 pandemic in our sample is shown in Figure 1.

Of the 17 pregnant women who accepted the flu vaccine, four subsequently did not perform the Tdap immunization because they had not received a recommendation from healthcare providers (4/4). Out of the 13 women who received the flu vaccine first and the Tdap later, 11 were recommended the pertussis immunization and two were aware that the vaccine protects their newborn from pertussis complications.

Of the 99 patients who performed the Tdap vaccine, 13 had previously received the flu vaccine and 86 had not. Out of the 86 pregnant women who accepted the pertussis vaccination after missing the flu vaccine, 69 chose to schedule this vaccine because they were recommended it, and 13 because they wanted to protect their offspring from pertussis complications. Out of the 96 women that have not received the Tdap vaccine, 30 (31.2%) stated that lockdown measures and fear of contracting COVID-19 reduced their access to vaccines. Of these women, 22 (73.3%) declared they would accept being vaccinated if the vaccine were administered during a prenatal visit/ hospitalization, and that the ideal place (19 answers, 63%) to be administered a vaccine is where routine antenatal visits are performed.

The main reasons to be vaccinated or to refuse immunization for each subgroup of patients are reported in Table 2, Table 3, Table 4, Table 5, Table 6 and Table 7. Both for the flu shot and Tdap, pregnant patients were more likely to accept the vaccines if they were recommended by a healthcare provider, whereas the main reason not to be vaccinated was the lack of such a recommendation.

Thirty-eight (19.5%) women received the recommendation for the flu vaccine, mainly from an obstetrician–gynecologist (23 women, 60.5%), and 28 women did not accept the vaccine despite the recommendation. The main reason not to be vaccinated in this group was that they did not think the vaccine was necessary for pregnant women/newborns (answer chosen 10 times). Seven women were administered the flu vaccine although they were not recommended it. The main reason to be vaccinated in this group was that they undergo such a vaccination every year. Indeed, to be vaccinated in the season 2018–2019 was consistently associated with being vaccinated the following year as well (*p* 0.0001). On the other hand, flu vaccination uptake in the previous season was not associated with a higher acceptance of Tdap in pregnancy (*p* 0.5152).

A total of 117 (60%) women stated they had been recommended the administration of the Tdap vaccine during pregnancy, mainly from an obstetrician–gynecologist (85 cases, 72.6%); 23 women did not accept the Tdap vaccine although they were recommended it. The main reasons not to be vaccinated in this group were that they did not think the vaccine was effective (chosen four times) or necessary (chosen four times) for pregnant women/newborns. Five women were administered the vaccine against pertussis although they were not recommended it. In this group, the main reason to be vaccinated was that they wanted to protect their babies against pertussis (chosen five times).

Considering only pregnant women recommended for flu and/or Tdap immunization, 73% (28 out of 38) refused the flu shot and 19% (23 out of 117) did not accept the administration of Tdap during pregnancy.

Considering the 28 patients who refused flu immunization despite the recommendation, 25 received the Tdap vaccine recommendation and among this group 18 (72%) accepted it.

With regard to the knowledge about the flu shot and Tdap, most women included in our study were aware that these vaccines protect the pregnant woman and the newborn from influenza and pertussis complications, and that they are harmless to the fetus (Table 8 and Table 9).

Ethnicity varied significantly among patients who received the influenza vaccine and those who did not (*p* 0.019), as well as those who received the Tdap vaccine and those who did not (*p* 0.0001). Table 10 and Table 11 contain detailed data on ethnic variations among individuals who received or refused influenza and Tdap vaccines.

## 4. Discussion

Pregnant women who gave birth in 2020 constitute a unique model to investigate whether and how the SARS-CoV-2 pandemic might have changed the approach toward maternal immunization. These women were expected to be recommended for and receive the flu vaccine during the seasonal influenza epidemic in the 2019–2020 winter (pre-pandemic) [9,22], and to be recommended for and receive the Tdap in their third trimester [8,23].

The population surveyed showed a low vaccine hesitancy rate and a high level of trust in healthcare providers, as demonstrated by the very low percentage of women that declared they were not willing to have their newborns vaccinated in adherence to the national schedules for immunization in childhood—less than 5%—and by the high proportion of women who declared that they rely on the obstetrician–gynecologist/midwife responsible for their prenatal care.

In the pre-COVID-19 era, less than 20% of the women in our sample were recommended the influenza vaccine, and only 9% were actually administered it. During the COVID-19 pandemic, in the midst of the first Italian lockdown, 60% of the women sampled were recommended to receive the Tdap vaccine, and more than 50% received it. It is extremely difficult to compare these percentages with pre-existing literature regarding vaccine uptake in pregnancy among Italian women due to the lack of a reliable national database. The percentage of Italian maternal immunization reported in the literature is very low. The influenza vaccination rate reported by Napolitano et al. [10] (2017) is about 10%, and D’Alessandro and co-authors [12] (2018) point out that only 1.4% of their sample were immunized against seasonal influenza. With regard to the Tdap vaccine, Marchetti et al. [11] (2018) reported that 52.2% of 600 pregnant women interviewed were willing to receive the vaccine. In the group analyzed by Vilca and co-authors [13] (2020), only 6.5% and 4.8% of the women were vaccinated against flu and pertussis, respectively. In our survey, the most striking piece of data is that in the same women before and after the coronavirus outbreak, the vaccination uptake increased more than fivefold. We explain such a substantial difference between the pre-COVID-19 and the COVID-19 era by a different sensitivity toward infectious disease complications and the preventive measures thereof.

Indeed, our survey shows that healthcare providers were more inclined to recommend the Tdap than the flu vaccine due to increased attention paid to communicable diseases. Another possible reason for this is that although maternal immunization is included in the agenda of the routine care of pregnancy, healthcare providers might have had less time to provide information in the pre-COVID-19 era due to busy schedules, whereas schedules with appointments far apart in the first Italian lockdown, i.e., allowing for more time between one patient and the following one in order to avoid crowding, made it possible to better inform patients on safety and the importance of maternal immunization. Lastly, obstetricians/gynecologists and midwives may feel insecure about recommending the flu vaccine in the first trimester, since they may erroneously fear teratogenicity at this gestational age.

The crucial role of healthcare providers in informing women about the safety and effectiveness of maternal immunization has been well reported in the literature [24,25,26]. Doctors/midwives should not only inform pregnant women as to the availability of the vaccines, but also provide them with information on possible pertussis and influenza complications, and how immunization can protect both the mother and the offspring; moreover, they should recommend vaccinations at the appropriate gestational age and schedule the immunization if the patient is willing to receive it [8,9].

Among those women who were recommended influenza and pertussis immunizations but who did not receive them, the belief that these were not necessary in pregnancy was the main reason not to follow the recommendation. These unfounded opinions underline the importance of correct and effective information provision on the topic. Information was, however, successful in the majority of cases of our sample, as demonstrated by the high number of correct answers about vaccine knowledge, higher than what was previously reported in the Italian population [11]. Belonging to a foreign ethnicity was associated with a lower likelihood to accept vaccinations despite recommendations, suggesting that linguistic barriers could have affected maternal immunization counselling. Other factors may be involved in the lower uptake of maternal immunization in foreign patients (lack of belief in vaccination, difficulty in returning to vaccine or antenatal care sites for appointment, or other cultural or socio-economic barriers). For this reason, it is important to organize specific training for obstetricians and midwives regarding the benefits and safety of maternal immunization, aiming to provide timely and coherent information to patients.

Concerns about vaccine safety were not a major issue in our sample population, unlike what is reported in the literature [27,28]. On the other hand, having been vaccinated in the previous years was positively associated with receiving the influenza vaccination in pregnancy. This was documented by other authors [24] as well, who therefore recommend to vaccinate non-pregnant young women for influenza in order to ensure that they will receive the vaccine when they become pregnant too. Despite the fact that some authors were concerned about a possible decrease in immunization rates [29,30] during the COVID-19 pandemic, our data demonstrate that vaccinations were perceived as an essential form of care. Those patients who declared that fear of contracting SARS-CoV-2 prevented them from scheduling their vaccination also stated they would accept being vaccinated during a consultation included in standard prenatal care. It is indeed reported in the literature that on-site vaccination is associated with a higher vaccination uptake [31], and that a strategy to improve access to vaccination is to have fewer appointments and to make the vaccination coincide with antenatal routine checks [32,33].

The limitations of our study are that only 37% of eligible women returned the questionnaire and took part in the survey, which could make the sample susceptible to selection biases (women with a higher vaccine uptake may be more willing to complete the questionnaire). Moreover, vaccine uptake was self-reported, hence misrecollections cannot be ruled out. The main strength of our paper is that we analyzed immunization uptake rates in the same set of patients before and after the COVID-19 outbreak.

The new challenge is COVID-19 vaccine immunization in pregnancy. COVID-19 vaccination administered in pregnancy provides both maternal and newborn passive immunization [34], as demonstrated from the transplacental passage of specific antibodies, similarly to what occurs in natural infection [35]. Preliminary data reported by Blakeway [36] about COVID-19 vaccine acceptance among pregnant patients revealed that less than one third of pregnant women accepted COVID-19 vaccination. The author’s conclusion points out the need for clear communication to improve awareness among pregnant women and healthcare professionals on vaccine safety, along with strategies to address vaccine hesitancy. Obstetrical staff should be trained to meet the need for precise information regarding the benefits, efficacy and safety of the COVID-19 vaccine as well as flu and Tdap vaccines in order to gain ethically and legally viable informed consent [37]. It is indeed likely that healthcare providers who are not sufficiently trained will not be able to obtain a satisfactory vaccine uptake, especially in a setting such as that of COVID-19 vaccination, in which hesitancy may be greater due to the perceived shortened testing time and the amplification of adverse effects in the public opinion [36]. A good level of training, awareness and involvement of the staff is therefore necessary in order to maximize vaccine uptake in the pregnant population [38].

## 5. Conclusions

Our survey has found out that the pregnant population do not always receive proper information regarding maternal immunization and its benefits and safety. Pregnant patients are more prone to accepting vaccination if the recommendation comes from obstetrical staff; therefore, counselling should become an integral part of pregnancy care. To be really effective, the vaccine proposal should come from well-trained staff and proper information should be given, also with the aim of obtaining legally and ethically valid consent.

Our survey also highlights how COVID-19 has put the threats posed by infectious diseases in the spotlight, thus creating an environment favorable to recommending vaccination for pregnant women. In order to increase the coverage for flu and Tdap immunization during pregnancy, recommendations should be offered and discussed by obstetrical staff, and vaccinations should be performed during routine prenatal care. Raising awareness in that regard will go a long way towards reducing COVID-19 vaccination hesitancy in the pregnant population.

In conclusion, the COVID-19 lesson has raised awareness regarding the role of vaccines in preventable diseases, positively changing attitudes toward immunization in pregnancy.

## Figures and Tables

**Table 1:** Sociodemographic characteristics.

**Age** (mean, SD)	35 (±15) years
**Parity**	*p* ≥ 1:106 (54.3%)
**Ethnicity**	
Italian	137 (70.2%)
Chinese	23 (11.8%)
Pakistani	7 (3.6%)
Other	28 (14.4%)
**Educational level**	
Primary school	38 (19.5%)
Secondary school	90 (46.2%)
Graduated, PhD	67 (34.3%)
**Employment status**	
Part-time–Full-time occupation	130 (66.7%)
Housewives	38 (19.5%)
Unemployed	27 (13.8%)

**Table 2:** Main reason to be vaccinated against influenza—patients who received the flu shot—17.

Answer	Number of Positive Answers
I was recommended to be vaccinated against influenza by a healthcare provider	9
I wanted to protect myself against influenza	3
I am vaccinated against influenza every year	5

**Table 3:** Main reason not to be vaccinated against influenza—patients who did not receive the flu shot—178.

Answer	Number of Positive Answers	
I was not recommended to be vaccinated against influenza by a healthcare provider	104	
I do not think the vaccine against influenza is effective for pregnant women/newborns	9	
	Recommendation from a healthcare provider	
	Yes	No
	5	4
I do not think the vaccine against influenza is safe for pregnant women/newborns	6	
	Recommendation from a healthcare provider	
	Yes	No
	2	4
I do not think the vaccine against influenza is necessary for pregnant women/newborns	19	
	Recommendation from a healthcare provider	
	Yes	No
	10	9
I had an adverse reaction in the past	2	
	Recommendation from a healthcare provider	
	Yes	No
	0	2
I was advised against this vaccine by a healthcare provider	4	
	Recommendation from a healthcare provider	
	Yes	No
	2	2
I was advised against this vaccine by friends	1	
	Recommendation from a healthcare provider	
	Yes	No
	0	1
I fear that the vaccine against influenza might harm the pregnancy	5	
	Recommendation from a healthcare provider	
	Yes	No
	1	4
I fear that the vaccine against influenza might harm the newborn	1	
	Recommendation from a healthcare provider	
	Yes	No
	0	1
I fear that the excipients of this vaccine might be harmful for the pregnancy	3	
	Recommendation from a healthcare provider	
	Yes	No
	0	3
I think I am not at risk for contracting influenza	2	
	Recommendation from a healthcare provider	
	Yes	No
	0	2
I think I have a healthy lifestyle and this protects me from diseases	3	
	Recommendation from a healthcare provider	
	Yes	No
	0	3
I think that breastfeeding gives enough protection against influenza to the baby	7	
	Recommendation from a healthcare provider	
	Yes	No
	0	7
Other reasons	12	
	Recommendation from a healthcare provider	
	Yes	No
	8	4

**Table 4:** Main reason to be vaccinated against pertussis—13 patients who accepted both flu and Tdap vaccines.

Answer	Number of Positive Answers
I was recommended to be vaccinated against pertussis by a healthcare provider	11
I wanted to protect my baby against pertussis	2

**Table 5:** Reasons not to be vaccinated against pertussis—4 patients who did not receive Tdap after being administered flu shot.

Answer	Number of Positive Answers
I was not recommended to be vaccinated against pertussis by a healthcare provider	4

**Table 6:** Main reason to be vaccinated against pertussis—86 patients who accepted Tdap after refusing flu shot.

Answer	Number of Positive Answers
I was recommended to be vaccinated against pertussis by a healthcare provider	69
I wanted to protect my baby against pertussis	13
I think that all pregnant women should be vaccinated against pertussis	4

**Table 7:** Main reason not to be vaccinated against pertussis—92 patients who refused both Tdap and flu shot.

Answer	Number of Positive Answers
I was not recommended to be vaccinated against pertussis by a healthcare provider	60
I do not think the vaccine against pertussis is effective for pregnant women/newborns	4
I do not think the vaccine against pertussis is safe for pregnant women/newborns	4
I do not think the vaccine against pertussis is necessary for pregnant women/newborns	5
I had an adverse reaction in the past	2
I was advised against this vaccine by a healthcare provider	1
I was advised against this vaccine by friends	1
I fear that the vaccine against pertussis might harm the pregnancy	1
I fear that the vaccine against pertussis might harm the newborn	1
I fear that the excipients of this vaccine might be harmful for the pregnancy	1
I think my newborn and I are not at risk for contracting pertussis	1
I contracted pertussis during childhood so I think my newborn and I are protected enough	3
I think I have a healthy lifestyle and this protects me from diseases	1
I think that breastfeeding gives enough protection against influenza to the baby	1
Other reasons	6

**Table 8:** Knowledge about the vaccine against influenza.

Answer	Number of Correct Answers
Flu shot protects the pregnant woman from influenza complications	146
Flu shot protects the newborn from influenza complications	103
Flu shot does not cause influenza in the newborn	131

**Table 9:** Knowledge about Tdap.

Answer	Number of Correct Answers
Pertussis might lead to severe complications in the newborn	160
Tdap protects the offspring against pertussis	150
Tdap does not cause pertussis in the newborn	149

**Table 10:** Flu refusal and acceptance according to different foreign ethnicities.

Ethnicity	Flu Shot Accepted	Flu Shot Refused
Chinese	0	23
Pakistani	0	7
Other	1	27

**Table 11:** Tdap refusal and acceptance according to different foreign ethnicities.

Ethnicity	Tdap Accepted	Tdap Refused
Chinese	5	18
Pakistani	1	6
Other	7	21

## Data Availability

The data presented in this study are available on request from the corresponding author. The data are not publicly available due to privacy reasons.

## Supplementary Materials

The following are available online at https://www.mdpi.com/article/10.3390/vaccines9101107/s1, File S1: Informed consent, File S2: Questionnaire.

## References

[1] Abu Raya B., Edwards K.M., Scheifele D.W., Halperin S.A. (2017). Pertussis and Influenza Immunisation during Pregnancy: A Landscape Review. Lancet Infect. Dis..

[2] Madhi S.A., Nunes M.C. (2018). Experience and challenges on influenza and pertussis vaccination in pregnant women. Hum. Vaccines Immunother..

[3] Giles M.L., Krishnaswamy S., Macartney K., Cheng A. (2019). The Safety of Inactivated Influenza Vaccines in Pregnancy for Birth Outcomes: A Systematic Review. Hum. Vaccines Immunother..

[4] Munoz F.M., Bond N.H., Maccato M., Pinell P., Hammill H.A., Swamy G.K., Walter E.B., Jackson L.A., Englund J.A., Edwards M.S. (2014). Safety and Immunogenicity of Tetanus Diphtheria and Acellular Pertussis (Tdap) Immunization during Pregnancy in Mothers and Infants: A Randomized Clinical Trial. JAMA.

[5] Laenen J., Roelants M., Devlieger R., Vandermeulen C. (2015). Influenza and Pertussis Vaccination Coverage in Pregnant Women. Vaccine.

[6] Vaccinazioni in Gravidanza: Proteggila per Proteggerli. Documento Congiunto SIGO, SIMP, AOGOI, AGUI, SITI, SIN, FNOPO, Rete Interaziendale Milano Materna Infantile (RIMMI), Vivere Onlus, Cittadinanzattiva 14 Marzo 2019. https://www.sigo.it/news/vaccinazioni-in-gravidanza-proteggila-per-proteggerli/.

[7] Piano Nazionale Prevenzione Vaccinale. https://www.salute.gov.it/portale/vaccinazioni/dettaglioContenutiVaccinazioni.jsp?lingua=italiano&id=4828&area=vaccinazioni&menu=vuoto.

[8] Position Paper. Nuove Sfide Nella Prevenzione per Mamma e Neonato. Investire Nelle Vaccinazioni Raccomandate in Gravidanza. La Pertosse. https://www.sigo.it/wp-content/uploads/2018/10/Position-Paper_PertosseF_26-10-18.pdf.

[9] Position Paper. Nuove Sfide Nella Prevenzione per Mamma e Neonato. Investire Nelle Vaccinazioni Raccomandate in Gravidanza. L’influenza. https://www.sigo.it/news/position-paper-e-sfide-nella-prevenzione-per-mamma-e-neonato-investire-nelle-vaccinazioni-raccomandate-in-gravidanza-linfluenza/.

[10] Napolitano F., Napolitano P., Angelillo I.F. (2017). Seasonal influenza vaccination in pregnant women: Knowledge, attitudes, and behaviours in Italy. BMC Infect. Dis..

[11] Marchetti F., Vilca L.M., Cetin I. (2019). Insights and expectations for Tdap vaccination of pregnant women in Italy. J. Matern. Fetal Neonatal. Med..

[12] D’Alessandro A., Napolitano F., D’Ambrosio A., Angelillo I.F. (2018). Vaccination Knowledge and Acceptability among Pregnant Women in Italy. Hum. Vaccin. Immunother..

[13] Vilca L.M., Cesari E., Tura A.M., Di Stefano A., Vidiri A., Cavaliere A.F., Cetin I. (2020). Barriers and Facilitators Regarding Influenza and Pertussis Maternal Vaccination Uptake: A Multi-Center Survey of Pregnant Women in Italy. Eur. J. Obstet. Gynecol. Reprod. Biol..

[14] Marinelli E., Busardò F.P., Zaami S. (2020). Intensive and pharmacological care in times of COVID-19: A “special ethics” for emergency?. BMC Med. Ethics.

[15] COVID-19 Vaccines While Pregnant or Breastfeeding. https://www.cdc.gov/coronavirus/2019-ncov/vaccines/recommendations/pregnancy.html.

[16] Society for Maternal-Fetal Medicine (SMFM) Statement: SARS-CoV-2 Vaccination in Pregnancy. https://www.smfm.org/publications/339-society-for-maternal-fetal-medicine-smfm-statement-sars-cov-2-vaccination-in-pregnancy.

[17] COVID-19 Vaccination Considerations for Obstetric–Gynecologic Care. Practice Advisory—December 2020. https://www.acog.org/clinical/clinical-guidance/practice-advisory/articles/2020/12/covid-19-vaccination-considerations-for-obstetric-gynecologic-care.

[18] COVID-19 Vaccines, Pregnancy and Breastfeeding. https://www.rcog.org.uk/en/guidelines-research-services/coronavirus-covid-19-pregnancy-and-womens-health/covid-19-vaccines-and-pregnancy/covid-19-vaccines-pregnancy-and-breastfeeding/.

[19] Position Paper ad Interim. Vaccinazione Anti COVID-19 e Gravidanza. https://www.sigo.it/wp-content/uploads/2021/01/VaccinoCovid19eGravidanza-SIGO-AOGOI-AGUI-AGITE-SIN_02-01-2021.pdf.

[20] Cavaliere A.F., Carabaneanu A.I., Perelli F., Matarrese D., Brunelli T., Casprini P., Vasarri P.L. (2020). Universal Screening for SARS-CoV-2 in Pregnant Women Admitted for Delivery: How to Manage Antibody Testing?. J. Matern. Fetal Neonatal. Med..

[21] Principi N., Esposito S. (2021). Is the Immunization of Pregnant Women against COVID-19 Justified?. Vaccines.

[22] American College of Obstetricians and Gynecologists (2018). ACOG committee opinion no. 732: Influenza vaccination during pregnancy. Obstet. Gynecol..

[23] Committee on Obstetric Practice Immunization and Emerging Infections Expert Work Group (2017). Committee Opinion No. 718: Update on Immunization and Pregnancy: Tetanus, Diphtheria, and Pertussis Vaccination. Obstet. Gynecol..

[24] Bartolo S., Deliege E., Mancel O., Dufour P., Vanderstichele S., Roumilhac M., Hammou Y., Carpentier S., Dessein R., Subtil D. (2019). Determinants of Influenza Vaccination Uptake in Pregnancy: A Large Single-Centre Cohort Study. BMC Pregnancy Childbirth.

[25] Lutz C.S., Carr W., Cohn A., Rodriguez L. (2018). Understanding Barriers and Predictors of Maternal Immunization: Identifying Gaps through an Exploratory Literature Review. Vaccine.

[26] Strassberg E.R., Power M., Schulkin J., Stark L.M., Mackeen A.D., Murtough K.L., Paglia M.J. (2018). Patient Attitudes toward Influenza and Tetanus, Diphtheria and Acellular Pertussis Vaccination in Pregnancy. Vaccine.

[27] Wilson R., Paterson P., Jarrett C., Larson H.J. (2015). Understanding factors influencing vaccination acceptance during pregnancy globally: A literature review. Vaccine.

[28] Chamberlain A.T., Seib K., Ault K.A., Orenstein W.A., Frew P.M., Malik F., Cortés M., Cota P., Whitney E.A.S., Flowers L.C. (2015). Factors Associated with Intention to Receive Influenza and Tetanus, Diphtheria, and Acellular Pertussis (Tdap) Vaccines during Pregnancy: A Focus on Vaccine Hesitancy and Perceptions of Disease Severity and Vaccine Safety. PLoS Curr..

[29] Saso A., Skirrow H., Kampmann B. (2020). Impact of COVID-19 on Immunization Services for Maternal and Infant Vaccines: Results of a Survey Conducted by Imprint—The Immunising Pregnant Women and Infants Network. Vaccines.

[30] Guidance on Routine Immunization Services during COVID-19 Pandemic in the WHO European Region, 20 March 2020. https://www.euro.who.int/en/health-topics/communicable-diseases/hepatitis/publications/2020/guidance-on-routine-immunization-services-during-covid-19-pandemic-in-the-who-european-region,-20-march-2020-produced-by-whoeurope.

[31] Hill L., Burrell B., Walls T. (2018). Factors influencing women’s decisions about having the pertussis-containing vaccine during pregnancy. J. Prim. Health Care.

[32] Maisa A., Milligan S., Quinn A., Boulter D., Johnston J., Treanor C., Bradley D.T. (2018). Vaccination against Pertussis and Influenza in Pregnancy: A Qualitative Study of Barriers and Facilitators. Public Health.

[33] Abu-Raya B., Maertens K., Edwards K.M., Omer S.B., Englund J.A., Flanagan K.L., Snape M.D., Amirthalingam G., Leuridan E., Damme P.V. (2020). Global Perspectives on Immunization During Pregnancy and Priorities for Future Research and Development: An International Consensus Statement. Front. Immunol..

[34] Collier A.-R.Y., McMahan K., Yu J., Tostanoski L.H., Aguayo R., Ansel J., Chandrashekar A., Patel S., Apraku Bondzie E., Sellers D. (2021). Immunogenicity of COVID-19 MRNA Vaccines in Pregnant and Lactating Women. JAMA.

[35] Cavaliere A.F., Marchi L., Aquilini D., Brunelli T., Vasarri P.L. (2021). Passive Immunity in Newborn from SARS-CoV-2-Infected Mother. J. Med. Virol..

[36] Blakeway H., Prasad S., Kalafat E., Heath P.T., Ladhani S.N., Le Doare K., Magee L.A., O’Brien P., Rezvani A., von Dadelszen P. (2021). COVID-19 Vaccination During Pregnancy: Coverage and Safety. Am. J. Obstet. Gynecol..

[37] Chervenak F.A., McCullough L.B., Bornstein E., Johnson L., Katz A., McLeod-Sordjan R., Nimaroff M., Rochelson B.L., Tekbali A., Warman A. (2021). Professionally Responsible Coronavirus Disease 2019 Vaccination Counseling of Obstetrical and Gynecologic Patients. Am. J. Obstet. Gynecol..

[38] Ogilvie G.S., Gordon S., Smith L.W., Albert A., Racey C.S., Booth A., Gottschlich A., Goldfarb D., Murray M.C.M., Galea L.A.M. (2021). Intention to Receive a COVID-19 Vaccine: Results from a Population-Based Survey in Canada. BMC Public Health.

