# Supplementary material for: Flu and Tdap Maternal Immunization Hesitancy in Times of COVID-19: An Italian Survey on Multiethnic Sample
Source: Vaccines (Basel). 2021 Sep 29;9(10):1107. doi: 10.3390/vaccines9101107 (PMC8537858; doi:10.3390/vaccines9101107)
Supplement: Supplementary file 1 [file vaccines-09-01107-s001.zip › vaccines-1373622-supplementary/Informed Consent.pdf]

INFORMATIVA E MANIFESTAZIONE DEL CONSENSO AL TRATTAMENTO DEI DATI PERSONALI (titolare trattamento Azienda USL Toscana Centro) (versione 1 del 01/06/2019)

关于个人信息处理的了解和意愿表达

托斯卡纳中部卫生局个人信息处理机构

（**2019/06/01** 第一版）

Sig. / ra ....<br>姓名<br>Nato / a a .... il ....<br>出生地点和日期

Finalità del trattamento. Titolare e responsabile del trattamento

L' Azienda USL Toscana Centro tratterà i Suoi dati personali in accordo alle responsabilità previste dalle norme di buona pratica clinica (d.l. 211/2003) ed alle disposizioni vigenti in materia di protezione dei dati personali (Regolamento UE n. 2016/679 (General Data Protection Regulation) in relazione al Questionario circa le Vaccinazioni in Gravidanza in Era COVID-19 che Le abbiamo chiesto di compilare.

个人信息处理的目的，权限和责任

托斯卡纳中部卫生局根据临床实践的规定(2003/211号法令）以及目前实施的个人信息保护条例（欧盟条例2016/679号《〈一般信息保护条例〉），对于我们所提出的，在冠状病毒病-19爆发期间，孕妇的疫苗接种情况的问卷当中所涉及到的个人信息进行处理。

La Dr.ssa Anna Franca Cavaliere è individuata quale responsabile del trattamento dei dati.
Anna Franca Cavaliere医生被确定为个人信息处理的责任人

La Dr.ssa Cavaliere si avvarrà dei collaboratori di seguito indicati, individuati quali incaricati del trattamento:
Cavaliere医生将会与以下人员合作，他们会被确定对信息的处理负责

Dr.ssa Laura Marchi, Dirigente Medico SOC Ostetricia e Ginecologia Santo Stefano- Azienda USL Toscana Centro
Laura Marchi 医生，托斯卡纳中部卫生局圣• 斯泰法诺妇产科主任医师

Ost. Claudia Foglia, Coordinatrice Reparto Ostetrico

Claudia Foglia 产科住院部护士长

I dati da lei conferiti saranno di tipo anagrafico ed anamnestico.
您所提供的信息将会被记录保存

Modalità del trattamento
处理方式

I dati saranno trattati mediante strumenti elettronici. I medici La identificheranno con un codice: i dati che La riguardano, raccolti nel corso dello studio, saranno registrati, elaborati e conservati per sette anni unitamente a tale codice. Soltanto il medico ed i soggetti autorizzati potranno collegare questo codice al Suo nominativo.

信息将使用电子设备处理。医生通过编号来区别数据提供者。对研究期间所收集的有关信息使用编号进行记录，整理以及保存7年。只有医生和被授权的人员才能通过编号连结到信息提供者的名字。

I dati potranno essere diffusi, ad esempio attraverso pubblicazioni scientifiche, statistiche e convegni scientifici, solo in forma rigorosamente anonima.

信息可能会通过科学出版，统计以及学术会议，以严格的不记名的的方式传播。

Potrà esercitare i diritti di cui all'art. 7 del Codice (es. accedere ai Suoi dati personali, integrarli, aggiornarli, rettificarli, opporsi al loro trattamento per motivi legittimi, ecc.) rivolgendosi direttamente alla Dr.ssa Cavaliere.

您可以直接与Cavaliere医生接洽，行使该法第7条所赋予的权利（查看您的个人信息，增加，修改，纠正，或者由于法律原因，反对他们对您的信息进行处理等等）

Consenso同意

Sottoscrivendo tale modulo acconsento al trattamento dei miei dati personali per gli scopi del Questionario nei limiti e con le modalità indicate nell'informativa fornitami con il presente documento.

回答这份问卷的问题表明我同意为着这些问题的用处，按上述所叙述的方式处理我个人的信息。

Firma dell’interessato ....
签字

Data　　　　　　　　　　　　　　　　日期
