# Supplementary material for: Flu and Tdap Maternal Immunization Hesitancy in Times of COVID-19: An Italian Survey on Multiethnic Sample
Source: Vaccines (Basel). 2021 Sep 29;9(10):1107. doi: 10.3390/vaccines9101107 (PMC8537858; doi:10.3390/vaccines9101107)
Supplement: Supplementary file 1 [file vaccines-09-01107-s001.zip › vaccines-1373622-supplementary/Questionnaire.pdf]

+

INTRODUZIONE AL QUESTIONARIO
前言

Gentile Signora
尊敬的夫人：

stiamo conducendo una ricerca riguardante la copertura vaccinale nelle donne in gravidanza e su quanto l’emergenza COVID-19 abbia influito sulle scelte delle gestanti in merito alle vaccinazioni routinarie.

我们这个调查是一项关于孕妇在怀孕期间对疫苗使用的普及情况以及新型冠状病毒病-19的爆发是否对孕妇疫苗接种有任何影响的研究工作。

I dati estrapolati dai questionari verranno elaborati a fini scientifici dalla Dr.ssa Anna Franca Cavaliere e dai suoi collaboratori.

回答问题所涉及到的各种信息，只用于Anna Franca Cavaliere 医生和她的合作同事进行科学研究。

Compilando il presente questionario, dichiara di aver preso visione e accettato l’informativa e manifestazione del consenso al trattamento dei dati personali allegata e di aver compreso gli scopi dello studio; inoltre, Lei è libera di non partecipare allo studio o di ritirarsi.

在回答如下问题的同时也表明她详细阅读并同意前面附件所说明的对个人信息的处理、使用以及清楚这些信息只作为科学研究的目的。另外每个人都可以自由地、不受任何影响地决定是否参加这项研究工作，而且可以更改自己的决定。

<u>Informazioni generali</u>

一般情况

1. Data di nascita (GG/MM/AAAA): ………./………./……….
   出生日期（日/月/年）

2. Composizione del nucleo familiare:
   家庭核心成员:
   - Partner配偶
   - Figli子女
   - Altro其他(specificare详细说明: ………………………………)

3. Se ha figli, quanti ne ha (escluso quello che aspetta)?: ………………..
   如果您有孩子，有几个（不包括未出生的）？

4. Se ha figli, ne indichi l’età:
   如果您有孩子，请写出他们的年龄:
   - Età figlio 1第一个: …………
   - Età figlio 2第二个: …………
   - Età figlio 3第三个: …………
   - Età figlio 4第四的: ……
   - Età figlio 5第五个: ……

5. Luogo di residenza户口所在地: …………………………

6. Nazionalità国籍: ………………………………….

7. Livello di istruzione学历:
   - Licenza scuola elementare小学毕业
   - Diploma di scuola media inferiore初中毕业
   - Diploma di scuola media superiore高中毕业
   - Laurea大学毕业
   - Master/Dottorato硕士/博士

8. Situazione lavorativa 工作情况:
   - Lavoratrice a tempo pieno (indicare professione: …………………………)
     全工（工作种类）
   - Lavoratrice part-time (indicare professione: ……………………………….)
     半工（工作种类）
   - Casalinga
     家庭妇女
   - Disoccupata
     失业

9. Stato civile:
   婚姻状况:
   - o Single单身
   - o Convivente一同居住
   - o Sposata结婚
   - o Vedova寡居

<u>Informazioni sullo stato di salute generale e sulla gravidanza</u>
怀孕期间和孕前身体的一般健康情况

1. Data dell'ultima mestruazione (GG/MM/AAAA): ………/………/………
   最后一次月经来潮的第一天（日/月/年）：

2. Data del parto (GG/MM/AAAA): ………/………/………
   （最小的）孩子的出生日期：日/月/年

3. Epoca gestazionale al parto: … settimane
   到分娩时，怀孕时间为...... 周（星期）

4. Numero di gravidanze complessive nella sua vita, compresa quella attuale e comprese eventuali interruzioni di gravidanza: ……….
   到现在一共怀孕次数（包括这一次和流产次数）........

5. Numero di parti vaginali ad oggi: ……….
   到现在一共顺产次数........

6. Numero di tagli cesarei ad oggi: ……….
   到现在一共剖腹产次数........

7. Gravidanza attuale当下的怀孕情况:
   - o Singola单胎
   - o Multipla (gemellare) 多胎（双胞胎）

8. Era affetta da patologie croniche prima della sua attuale gravidanza (diabete, ipertensione…)?
   怀孕之前是否有慢性疾病（糖尿病，高血压......）？
   - o Sì有
   - o No没有

   Se ha risposto sì, quale nello specifico? ……………………………………
   如果有，是哪一种？........

9. Le è stata diagnosticata qualche patologia durante la gravidanza?
怀孕期间是否有检查出某种疾病？

- o Ipertensione高血压
- o Diabete gestazionale孕期高血糖
- o Ipotiroidismo甲减（甲状腺机能低下）
- o Placenta previa胎盘前置
- o Altro其它(specificare详细说明：……………………………….)

10. Nel corso della gravidanza è mai stata ricoverata in ospedale per accertamenti/problemi/complicanze)?
怀孕期间有没有因为不正常情况而住过医院？

- o Sì有
- o No没有

Se ha risposto sì, specificare le problematiche insorte e quando (specificare l'ultimo ricovero): ……………………

若果有，请详细说明住院的原因以及最后一次住院的时间

11. Quante visite ostetriche e prenatali ha effettuato durante la gravidanza attuale? ………………………………………………

这一次怀孕期间，作过几次产科检查和产前检查？

12. Da quale figura professionale è stata principalmente seguita durante la gravidanza attuale? Può selezionare più di una risposta:
这次怀孕期间的各种检查主要是由哪方面医生进行的，可以有多项选择：

- o Ginecologo妇科医生
- o Ostetrico产科医生
- o Medico di base家庭医生
- o Altro professionista sanitario其他专业医生

13. Dove si è recata principalmente per essere seguita nel corso dell'attuale gravidanza? Può selezionare più di una risposta:
这次怀孕期间的检查主要是在哪一种医疗卫生单位进行的？

- o Ospedale医院
- o Studio medico (libera professione) 私人诊所（专家个人门诊）
- o Consultorio城区门诊
- o Poliambulatorio综合门诊
- o Altro其它(specificare详细说明：………………………….)

14. Peso pre-gravidanza: ………. kg
怀孕前的体重............... 公斤

15. Peso attuale: ………. kg
现在的体重........   ...公斤

16. Altezza: ………. cm
身高：........   ....厘米

Vaccinazioni

疫苗接种情况

1. Ha ricevuto la vaccinazione ANTINFLUENZALE più recente (stagione 2019/2020)?
   您最近（2019/2020）是否接种过抗流感疫苗？
   - Sì有
   - No没有

2. Se ha ricevuto la vaccinazione antinfluenzale in questa gravidanza, ha trovato delle difficoltà nel farsi somministrare il vaccino?
   如果在这次怀孕期间您接种过抗流感疫苗，为了接种疫苗您是否遇到过困难？
   - Sì有(quali哪些困难?……………………………………………………..)
   - No 没有
   -
3. Ha ricevuto la vaccinazione antinfluenzale nella stagione 2018/2019?
   您在2018/2019期间是否接种过抗流感疫苗？
   - Sì 有
   - No没有

4. Nel corso della sua attuale gravidanza, qualcuno degli operatori sanitari con cui è entrata in contatto le ha raccomandato di vaccinarsi contro l'influenza? (oltre alla raccomandazione contenuta nel Libretto di Gravidanza della Regione Toscana)
   在这次怀孕期间，您所接触到的医护人员中是否有人建议您接种抗流感疫苗（托斯卡纳大区所发的孕妇手册中建议之外的）
   - Sì有
   - No没有

   Se sì, quale figura professionale le ha raccomandazione la vaccinazione antinfluenzale? Può selezionare più di una risposta:
   如果有，是哪方面的人员建议您接种抗流感疫苗的？可以多选。
   - Ginecologo妇科医生
   - Ostetrico产科医生
   - Medico di base家庭医生
   - Pediatra儿科医生
   - Farmacista药剂师
   - Altro 其他(specificare请说明……………………………………………….)

Se sì, le sono state fornite indicazioni chiare e precise riguardo alla vaccinazione (efficacia, sicurezza, dove acquistarla, dove praticarla…)?
如果有，他们是否清楚、准确地解释了接种疫苗的信息（疫苗的功效、安全性，在哪里可以得到疫苗以及可以在哪里接种）？

- Sì有
- No没有

5. Ha ricevuto la vaccinazione ANTIPERTOSSE (dTpa) durante la sua attuale gravidanza?
   这次怀孕您是否接种过百日咳疫苗（百白破三价苗）
   - Sì 有
   - No没有
6. In quale sede è stata effettuata la vaccinazione antipertosse?
   您在哪里接种的百日咳疫苗？
   - Centro vaccinale 疫苗接种中心
   - Ospedale医院
   - Consultorio城区诊所
   - Poliambulatorio综合门诊部
   - Studio del medico di base家庭医生诊所
   - Studio del ginecologo libero professionista妇科专家门诊
   - Altro其他(specificare说明：……………………………………)

7. Se ha ricevuto la vaccinazione antinfluenzale in questa gravidanza, ha trovato delle difficoltà nel farsi somministrare il vaccino?
   如果您接种过百日咳疫苗，整个过程您是否遇到过任何困难？
   - Sì有(quali什么困难？……………………………………………………….)
   - No没有

8. Nel corso della sua attuale gravidanza, qualcuno degli operatori sanitari con cui è entrata in contatto le ha raccomandato di vaccinarsi contro la pertosse?
   在这次怀孕期间，您所接触到的医护人员中是否有人建议您接种百日咳疫苗？
   - Sì有
   - No没有

   Se sì, quale figura professionale le ha raccomandazione la vaccinazione antipertosse? Può selezionare più di una risposta:
   如果有，是哪一方面的人员建议您接种的（可以多选）？
   - Ginecologo妇科医生
   - Ostetrico产科医生
   - Medico di base家庭医生
   - Pediatra儿科医生
   - Farmacista药剂
   - Altro其他(specificare说明……………………………………………….)

Se sì, le sono state fornite indicazioni chiare e precise riguardo alla vaccinazione (efficacia, sicurezza, dove acquistarla, dove praticarla…)?

如果有，他们是否清楚、准确地解释了接种疫苗的信息（疫苗的功效、安全性，在哪里可以得到疫苗以及可以在哪里接种）？

- Sì有
- No没有

Conoscenza delle vaccinazioni durante la gravidanza

对怀孕期间接种疫苗的了解

Indichi se, secondo lei, tali affermazioni sono vere o false:

在您看来，下面的说法正确还是错误：

	对 V	错 F
La vaccinazione antinfluenzale durante la gravidanza protegge le gravide dalle complicanze associate all'influenza.<br>怀孕期间接种抗流感疫苗可以防止流感对孕妇的影响。	○	○
Il vaccino antinfluenzale è efficace nel prevenire le infezioni collegate al virus dell'influenza nei neonati nei primi mesi di vita<br>流感疫苗可以使新生儿在最初的几个月里有效地预防流感病毒	○	○
Il vaccino antinfluenzale può, successivamente alla somministrazione, provocare l'influenza nella mamma e nel feto.<br>接种抗流感疫苗之后，可能引起妈妈和新生儿得流行性感冒	○	○
La pertosse può essere una malattia grave nei bambini soprattutto nei primi mesi di vita<br>百日咳对新生儿在最初的几个月内是一种很重的疾病	○	○
La vaccinazione antipertosse durante la gravidanza protegge dalla patologia il neonato durante i primi mesi di vita.<br>怀孕期间接种百日咳疫苗可以预防新生儿在最初的几个月内得病	○	○
Il vaccino antipertosse può, successivamente alla somministrazione, provocare la pertosse nella mamma e nel feto.<br>接种百日咳疫苗之后，可能会使妈妈和新生儿患百日咳病。	○	○

1. Se durante la sua attuale gravidanza HA EFFETTUATO la vaccinazione ANTIFLUENZALE, indichi le principali motivazioni per cui l'ha fatto (può selezionare più di una risposta):
如果您在怀孕期间接种了抗流感疫苗，请说一下您这样做的主要想法是什么（可以多选）？

- o La vaccinazione mi è stata raccomandata da un operatore sanitario
  医护人员建议我做的
- o Voglio proteggere me stessa
  为了保护我自己
- o Voglio proteggere il mio bambino
  为了保护我的孩子
- o Penso che tutte le donne incinte dovrebbero farlo
  我想所有的孕妇都应该接种
- o Effettuo annualmente il vaccino antinfluenzale
  我每年都接种抗流感疫苗
- o Parenti/amici/conoscenti mi hanno consigliato di farlo
  亲人/朋友/熟人建议我做的
- o Altro其他：…………………………………………………………………….

2. Se durante la sua attuale gravidanza NON ha effettuato la vaccinazione ANTINFLUENZALE, indichi le principali motivazioni per cui non l'ha fatto (può selezionare più di una risposta):
如果怀孕期间没有接种抗流感疫苗，您的主要想法是什么（可以多选）？

- o La vaccinazione non mi è stata raccomandata
  没有人建议我接种
- o Non credo che questo vaccino sia efficace per le gravide/per il feto
  我认为这个疫苗对孕妇和孩子没有效果
- o Non credo che questo vaccino sia sicuro per le gravide/per il feto
  我认为这种疫苗对孕妇和孩子不安全
- o Non credo che questo vaccino sia necessario per le gravide/per il feto
  我不认为这种疫苗对孕妇和孩子是必要的
- o Ho avuto una reazione avversa in passato
  我过去接种疫苗的时候有过反应
- o Mi è stato sconsigliato da un professionista sanitario
  医护人员建议我不要做
- o Mi è stato sconsigliato da parenti/amici/conoscenti
  我的亲人/朋友/熟人建议我不要接种
- o Ho paura che questo vaccino possa essere dannoso per la gravidanza
  害怕接种疫苗对怀孕有不好的影响

- Ho paura che questo vaccino possa essere dannoso per il feto
  害怕疫苗对孩子有伤害
- Temo che alcuni eccipienti del vaccino siano dannosi per il feto
- Non ritengo che io/il mio bambino siamo a rischio di contrarre questa patologia, e comunque non in forma grave
  担心疫苗中的其它成分会对孩子有害。我不认为我和孩子会得这种病，至少不会很严重
- Ho uno stile di vita sano e ciò mi protegge dalle malattie infettive
  我有很健康的生活习惯，所以我能抵御传染病
- Ritengo che l’allattamento conferisca al bambino immunità sufficiente
  我认为母乳喂养会使孩子有足够的抵抗力
- Altro其它: ……………………………………………………………………………

3. Se durante la sua attuale gravidanza HA EFFETTUATO la vaccinazione ANTIPERTOSSE, indichi le principali motivazioni per cui l’ha fatto (può selezionare più di una risposta):
   如果您在怀孕期间接种过百日咳疫苗，请说一下您的主要想法（可多选）
   - La vaccinazione mi è stata raccomandata da un operatore sanitario
     医护人员的建议
   - Voglio proteggere me stessa
     为了保护我自己
   - Voglio proteggere il mio bambino
     为了保护我的孩子
   - Penso che tutte le donne incinte dovrebbero farlo
     认为所有的孕妇都应该接种
   - Parenti/amici/conoscenti mi hanno consigliato di farlo
     亲人/朋友/熟人建议我接种
   - Altro其他: ……………………………………………………………………
4. Se durante la sua attuale gravidanza NON ha effettuato la vaccinazione ANTIPERTOSSE, indichi le principali motivazioni per cui non l’ha fatto (può selezionare più di una risposta):
   如果怀孕期间您没有接种百日咳疫苗，请说一下你的主要想法
   - La vaccinazione non mi è stata raccomandata
     没有人建议我要接种
   - Non credo che questo vaccino sia efficace per le gravide/per il feto
     我不认为这个疫苗对孕妇和孩有效果
   - Non credo che questo vaccino sia sicuro per le gravide/per il feto
     我不认为疫苗对孕妇和孩子是安全的
   - Non credo che questo vaccino sia necessario per le gravide/per il feto
     我不认为这个疫苗对孕妇和孩子是必要的
   - Ho avuto una reazione avversa in passato
     过去接种疫苗的时候有过反应

- o Mi è stato sconsigliato da un professionista sanitario
  医护人员建议我不要做
- o Mi è stato sconsigliato da parenti/amici/conoscenti
  亲人/朋友/熟人建议我不要做
- o Ho paura che questo vaccino possa essere dannoso per la gravidanza
  怕这个疫苗对怀孕有害处
- o Ho paura che questo vaccino possa essere dannoso per il feto
  怕这种疫苗对孩子有伤害
- o Temo che alcuni eccipienti del vaccino siano dannosi per il feto
  担心疫苗内的其它成分对孩子不好
- o Non ritengo che io/il mio bambino siamo a rischio di contrarre questa patologia, e comunque non in forma grave
  不认为我以及孩子会得这种病，至少不会很重
- o Ho avuto la pertosse nell’infanzia quindi ritengo di essere immune
  我小的时候得过百日咳，所以我应该有抗体了
- o Ho uno stile di vita sano e ciò mi protegge dalle malattie infettive
  我有一个健康的生活方式，所以不会得传染病
- o Ritengo che l’allattamento conferisca al bambino immunità sufficiente
  我认为母乳喂养会使孩子得到抵抗力
- o Altro其它: ……………………………………………………………………………………

5. Se NON è stata vaccinata durante questa gravidanza, lei avrebbe preso in considerazione la possibilità di vaccinarsi se qualche operatore sanitario glielo avesse raccomandato?
如果这次怀孕您还没有接种疫苗，而有人建议您接种，您会去做吗？

- o Sì会
- o No不会
- o Non lo so不知道
- o Altro其它: ……………………………………………………………………………

6. Se NON è stata vaccinata durante questa gravidanza, quanto pensa che abbia influito il lock-down dovuto al COVID-19 e la raccomandazione all’isolamento sociale sulla sua scelta?
如果这次怀孕期间，您没有接种疫苗，冠状病毒-19爆发期间，封城禁足令对您的影响有多大？

- o Per nulla一点也没有
- o Poco有点
- o Abbastanza影响挺大
- o Molto非常大

7. Se NON è stata vaccinata durante questa gravidanza, pensa che lo avrebbe fatto se la vaccinazione le fosse stata offerta contestualmente ad un appuntamento per una visita/ecografia di controllo/ricovero?
如果这次怀孕您没有接种疫苗，在约好的检查、B超、或者住院的时候，为您提供接种疫苗，您会接受吗？

- o Sì会
- o No不会

8. Ha intenzione di vaccinare suo figlio secondo il calendario vaccinale previsto dal Ministero della Salute?
您会按照国家卫生部的要求，按时给您的孩子接种疫苗吗？

- o S ì 会
- o No不会
- o Non lo so不知道

9. Indichi l'influenza che ciascuna delle figure professionali indicate ha avuto sulla sua decisione di vaccinarsi durante la sua gravidanza:
对于您是否接种疫苗，下列人员对您做决定的影响程度

		1	2	3	4	5	
Ginecologo* 妇科医生	Poca influenza 影响小	O	O	O	O	O	Molta influenza 影响大
Ostetrica* 产科医生	Poca Influenza 影响小	O	O	O	O	O	Molta influenza 影响大
Medico di base* 家庭医生	Poc influenza 影响小	O	O	O	O	O	Molta influenza 影响大
Pediatra* 儿科医生	Poca influenza 影响小	O	O	O	O	O	Molta influenza 影响大
Farmacista* 药剂师	Poca influenza 影响小	O	O	O	O	O	Molta influenza 影响大

10. Ci sono altre figure non citate che lei ha ritenuto molto influenti su questa sua decisione? Specificare: ……………………………………………………
有没有上述没提到的人员对于您的决定有过影响？请说明：

11. Come considera queste fonti di informazione riguardo le vaccinazioni in gravidanza?
对以 下信息来源，有关怀孕期间接种疫苗的说法，您的看法是什么？

	Niente Affidabile 不可信	Poco affidabile 有些可信	Abbastanza affidabile 相当可信	Molto affidabile 非常可信	Completamente affidabile 完全可信
Professionista sanitario responsabile del Controllo prenatale (ginecologo/ostetriche) 孕期检查的负责医生（妇科医生/产科医生	○	○	○	○	○
Altri medici (medico di base/pediatra) 其他医生（家庭医生/儿科医生）	○	○	○	○	○
Altri professionisti sanitari (infermiere/farmacista) 其他医务人员（护士/药剂师）	○	○	○	○	○
Informazione ufficiale del Ministero di Sanità o Autorità Sanitarie nazionale 国家卫生部的官方信息或者医疗卫生主管部门	○	○	○	○	○
Internet(blogs, forum pagine web non ufficiali) 互联网（非官方的博客，论坛，网页）	○	○	○	○	○
Social media (Facebook, Instagram, Twitter) 社会媒体（脸书，即时电报，推特）	○	○	○	○	○
Partner/parenti/amici 配偶/亲人/朋友	○	○	○	○	○
Altre fonti d'informazione 其它信息来源	○	○	○	○	○

12. Se potesse scegliere il posto ideale per ricevere le vaccinazioni durante la gravidanza, quale sarebbe? Può selezionare più di una risposta:

如果可以选择，您认为在哪个地方接种疫苗比较好（可以多选）？

- o Nello stesso posto in cui si svolgono le visite di controllo prenatale (ospedale, ambulatorio怀孕期间检查的地方（医院/门诊….)
- o Durante il corso di preparazione alla nascita准备生小孩的那段时间里
- o Dal medico di base在家庭医生那里
- o Al centro vaccinale在疫苗接种中心
- o In farmacia在药店
- o Altro其它：………………………………………………………………………….)
